# Editorial: The legacy of Dr. Roger W. Sperry: current advances in brain lateralization and interhemispheric transfer

**DOI:** 10.3389/fnhum.2024.1433410

**Published:** 2024-05-29

**Authors:** Dahlia W. Zaidel, Mara Fabri

**Affiliations:** ^1^Department of Psychology, University of California, Los Angeles, Los Angeles, CA, United States; ^2^Dipartimento di Scienze della Vita e dell'Ambiente, Università Politecnica delle Marche, Ancona, Italy

**Keywords:** Nobel Prize, hemispheric specialization, commissurotomy, split-brain, consciousness, callosotomy, disconnection syndrome

Roger W. Sperry's Nobel Prize for Physiology or Medicine in 1981 (shared with David Hubel and Torsten Wiesel) was awarded for elucidating functional specialization in the left and right cerebral hemispheres of commissurotomy cases studied at Caltech, Pasadena (Sperry, 1968, 1974). For the first time, scientific light with far reaching implications was shed on the nature of hemispheric specialization, consciousness, and inter-hemispheric communication in the human brain. This followed Sperry (1961, 1964) early long quest to unravel corpus callosum functions, the major inter-hemispheric communication route. Commissurotomy, known in lay terms as “split-brain,” alleviated pharmacologically intractable severe epilepsy (Bogen and Vogel, 1962; Bogen, 1985). The findings in Sperry's lab inspired myriad neuroscientific research into consciousness (Sperry et al., 1979; Koch et al., 2016; Mashour and Hudetz, 2018), functional compensation following hemispheric brain injury (Riès et al., 2016), right hemisphere language (Zaidel, 1976; Corballis, 2014), plasticity of language development (Martin et al., 2022), neuroanatomy and morphology of callosal fibers (Aboitiz et al., 2003; Mooshagian, 2008; Fame et al., 2011), nature of hemispheric specialization in the healthy brain (Esteves et al., 2020; Hartwigsen et al., 2021), handedness (Sha et al., 2021), and much more.

Importantly, the discoveries by Sperry's group were based on right-handed cases who underwent a single-stage (same day), complete surgical section of the forebrain commissures, which include the corpus callosum, anterior, and hippocampal commissures, enabling investigations into language, perception, cognition, and consciousness in each disconnected hemisphere (Sperry, 1974; Sperry et al., 1979). In all of these cases, the main language centers were lateralized to the left hemisphere. In contrast, patients who underwent partial commissurotomy where the splenium portion of the corpus callosum was left intact, also studied by Sperry, showed absence of the hemispheric disconnection syndrome (Bogen, 1993).

Research centers in other countries applied callosal disruption surgery to treat drug-resistant epilepsy. Since the 1980's, increased number of epilepsy patients underwent partial or total callosotomy (sectioning only the corpus callosum) in Ancona, Italy, where neurosurgeon Isacco Papo performed the surgery at the Ancona Hospital. Studying these patients presented a unique opportunity in the past 40 years to continue investigations into the role of the corpus callosum in interhemispheric communication. Research on patients with various degrees of callosotomy have clarified results mainly relating to simple sensory or motor functions, specifically which callosal routes facilitate transfer different kinds of sensory and motor information between the two hemispheres. These findings with the Ancona group of patients has been described and summarized (Fabri et al., 2017; Fabri and Polonara, 2023).

Topics in the foregoing are addressed in the articles published in this Research Topic honoring Roger W. Sperry.

Sperry's scientific contributions and remarkable research thrust are reviewed in detail in the Berlucchi and Marzi article. They describe the series of critical, original questions coupled with creative methodology that have led Sperry on a path where he investigated neuronal connectivity, chemoaffinity, consciousness, corpus callosum functionality, and hemispheric specialization in complete commissurotomy patients. From investigations in animals to human studies, in each one of these research fields he contributed critical discoveries that advanced science immeasurably. The Berlucchi and Marzi paper provides background and insights to the uniqueness of his thinking and his research approach.

Basic brain and neuropsychological research informs clinical applications. Christensen et al.'s study applied interhemispheric transfer time (IHTT) to asses extent of brain damage following head concussion in children and adolescents. This clinical study sheds light on the consequences of such condition to callosal functionality. It benefitted from Roger Sperry's extensive work on the functions of the corpus callosum in both animals and the human commissurotomy patients.

Salillas et al. addressed the issue of math functions in the left and right hemispheres. The findings with the commissurotomy patients made by Sperry's group at Caltech (Sperry, 1974) revealed that simple mathematical calculation specialization in the disconnected left hemisphere was consistent with unilateral lesion studies showing acalculia symptoms in patients with left hemisphere injury, that is, that the left hemisphere is superior to the right in arithmetical calculation. But Sperry's findings also showed for the first time some calculation ability, albeit limited, in the disconnected right hemisphere as well. In their important article, Salillas et al. go further, they provide comprehensive review of brain lateralization of math functions in neurological patients as well as in healthy adults and children, substantially extending and clarifying the hemispheric role in arithmetic. Their work revealed a greater contribution by the right hemisphere and the suggestion that normally both hemispheres participate in arithmetical calculation, particularly through parietal lobe regions.

The study by Mazzi et al. was carried out in adult healthy volunteers and investigated the whole pattern of intra- and inter-hemispheric cortico-cortical connectivity changes induced by low frequency repetitive transcranial stimulation (rTMS) applied over the right posterior parietal cortex (rPPC). The results revealed a significant increase in coherence in delta, theta, alpha and beta frequency bands between rPPC and the contralateral homologous sites, indicating an excitatory connectivity. An increase in the same frequency bands was also found between rPPC and right frontal sites, reflecting the activation of the fronto-parietal network within the right hemisphere. The authors conclude that subthreshold rTMS applied to the right posterior parietal cortex modulated functional connectivity and coherence in the brain. Moreover, the results further confirm previous evidence indicating that the increase of coherence values is related to intra- and inter-hemispheric inhibitory effects of rTMS. This observation challenges the notion of reciprocal inhibition between the hemispheres and suggests a role for maladaptive plasticity in spatial neglect. Implications for devising evidence-based rehabilitation protocols following stroke are also pointed out.

Marcantoni et al. investigated interhemispheric functional connectivity (FC) in the Ancona group of callosotomized patients by using functional magnetic resonance imaging (fMRI). Functional connectivity is defined in terms of temporal correlations between physiological signals from cortical areas anatomically separated, and it mainly depends upon structural connectivity. Interhemispheric FC appears mostly supported by the corpus callosum (CC), although the available evidence are not conclusive. The study by Marcantoni et al. was carried out in a few patients who have undergone partial or total surgical resection of the CC. The results show that the interhemispheric FC is generally preserved between primary sensory cortical areas, but often reduced or lacking between the associative areas. The authors suggest that residual FC observed in the absence of the CC might be supported through subcortical extra-callosal pathways.

Pinto et al. compared deliberate vs. automatic processing in visual integration across the left and right hemispheres, starting from recent evidence that the Ancona split-brain patients can detect and localize stimuli anywhere in the visual field, both verbally and with either hand. The authors explored this cross-hemifield interaction in detail with a single complete callosotomy patient, albeit undergoing multi-stage surgery (not all callosal fibers sectioned the same day). The results suggest to the authors that when the patient was forced to adopt a conscious and deliberate approach, processing seemed to be unified across the left and right visual fields, and thus across the hemispheres. The authors offer a two-route explanation: in healthy subjects, the visual information from the two hemifields is normally integrated in an automatic, unconscious fashion via the intact splenium. In this split-brain patient, in whom the splenium was severed, some information transfer remained. Therefore, a second route, perhaps less visual and more symbolic, may become functional when the patient is forced to use a deliberate, consciously controlled approach. This two-route model raises issues that could help clarify some of the controversial issues in split-brain research.

Studies carried out with the Ancona group of split-brain patients have provided much important information, including the observation that they have developed compensatory strategies to solve conflicting situations in everyday life. Such strategies could potentially lead to a misinterpretation of split-brain performance results. On the whole, this research field has provided a great opportunity to study lateralized and bilaterally represented brain functions as well as extra-callosal pathways facilitating hemispheric cooperation.

Multiple issues addressing hemispheric specialization and functional laterality remain to be studied further, particularly with regards to higher cognitive functions such as thinking and problem solving, conceptualizing, learning, imagining, emotions, memory, attention, and consciousness.

## Author contributions

DZ: Writing – original draft, Writing – review & editing. MF: Writing – original draft, Writing – review & editing.

## References

[B1] Aboitiz F. López J. Montiel J. (2003). Long distance communication in the human brain: timing constraints for inter-hemispheric synchrony and the origin of brain lateralization. Biol. Res. 36, 89–99. 10.4067/S0716-97602003000100007 12795208

[B2] Bogen J. E. (1985). “Split-brain syndromes,” in Handbook of Clinical Neurology, Vol 45, eds. P. Vinken, and G. Bruyn (Oxford: Oxford University Press), 99–106.

[B3] Bogen J. E. (1993). “The callosal syndromes,” in Clinical Neuropsychology, 3rd ed., eds. K. M. Heilman, and E. Valenstein (New York, NY: Oxford University Press), 337–407.

[B4] Bogen J. E. Vogel P. J. (1962). Cerebral commissurotomy in man. Preliminary case report. Bull. Los Angeles Neuro. Soc. 27, 169–172.

[B5] Corballis M. C. (2014). Left brain, right brain: facts and fantasies. PLoS Biol. 12:e1001767. 10.1371/journal.pbio.1001767 24465175 PMC3897366

[B6] Esteves M. Lopes S. S. Almeida A. Sousa N. Leite-Almeida H. (2020). Unmasking the relevance of hemispheric asymmetries-Break on through (to the other side). Prog. Neurobiol. 192:101823. 10.1016/j.pneurobio.2020.101823 32433927

[B7] Fabri M. Foschi N. Pierpaoli C. Polonara G. (2017). “Split-brain human subjects,” in Lateralized Brain Functions: Methods Human and Non-Human Species, eds. L. Rogers, and G. Vallortigara (Totowa, NJ: Humana Press), 29–78. 10.1007/978-1-4939-6725-4_2

[B8] Fabri M. Polonara G. (2023). Functional topography of the corpus callosum as revealed by fMRI and behavioural studies of control subjects and patients with callosal resection. Neuropsychologia 183:108533. 10.1016/j.neuropsychologia.2023.108533 36906223

[B9] Fame R. M. MacDonald J. L. Macklis J. D. (2011). Development, specification, and diversity of callosal projection neurons. Tren. Neurosci. 34, 41–50. 10.1016/j.tins.2010.10.002 21129791 PMC3053014

[B10] Hartwigsen G. Bengio Y. Bzdok D. (2021). How does hemispheric specialization contribute to human-defining cognition? Neuron 109, 2075–2090. 10.1016/j.neuron.2021.04.024 34004139 PMC8273110

[B11] Koch C. Massimini M. Boly M. Tononi G. (2016). Neural correlates of consciousness: progress and problems. Nat. Rev. Neurosci. 17, 307–321. 10.1038/nrn.2016.22 27094080

[B12] Martin K. C. Ketchabaw W. T. Turkeltaub P. E. (2022). Plasticity of the language system in children and adults. Handb. Clin. Neurol. 184, 397–414. 10.1016/B978-0-12-819410-2.00021-7 35034751 PMC10149040

[B13] Mashour G. A. Hudetz A. G. (2018). Neural correlates of unconsciousness in large-scale brain networks. Tren. Neurosci. 41, 150–160. 10.1016/j.tins.2018.01.003 29409683 PMC5835202

[B14] Mooshagian E. (2008). Anatomy of the corpus callosum reveals its function. J. Neurosci. 28, 1535–1536. 10.1523/JNEUROSCI.5426-07.2008 18272674 PMC6671538

[B15] Riès S. K. Dronkers N. F. Knight R. T. (2016). Choosing words: left hemisphere, right hemisphere, or both? Perspective on the lateralization of word retrieval. Ann. N Y Acad. Sci. 1369, 111–131. 10.1111/nyas.12993 26766393 PMC4874870

[B16] Sha Z. Pepe A. Schijven D. Carrión-Castillo A. Roe J. M. Westerhausen R.. (2021). Handedness and its genetic influences are associated with structural asymmetries of the cerebral cortex in 31,864 individuals. Proc. Natl. Acad. Sci. U S A. 118:e2113095118. 10.1073/pnas.2113095118 34785596 PMC8617418

[B17] Sperry R. W. (1961). Cerebral organization and behavior. Science 133, 1749–1757. 10.1126/science.133.3466.1749 17829720

[B18] Sperry R. W. (1964). The great cerebral commissure. Sci. Am. 210, 42–52. 10.1038/scientificamerican0164-42 14088562

[B19] Sperry R. W. (1968). Hemisphere deconnection and unity in conscious awareness. Am. Psychol. 23, 723–733. 10.1037/h0026839 5682831

[B20] Sperry R. W. (1974). “Lateral specialization in the surgically separated hemispheres,” in Neuroscience Third Study Program, Vol. 3, eds. F. O. Schmitt, and F. G. Worden (Cambridge: MIT Press), 5–19.

[B21] Sperry R. W. Zaidel E. Zaidel D. (1979). Self-recognition and social awareness in the deconnected minor hemisphere. Neuropsychologia 17, 153–166. 10.1016/0028-3932(79)90006-X 379688

[B22] Zaidel E. (1976). Auditory vocabulary of the right hemisphere following brain bisection or hemidecortication. Cortex 12, 191–211. 10.1016/S0010-9452(76)80001-9 1000988

